# Interpreter-mediated interactions between people using a signed respective spoken language across distances in real time: a scoping review

**DOI:** 10.1186/s12913-022-07776-y

**Published:** 2022-03-24

**Authors:** Camilla Warnicke, Sarah Granberg

**Affiliations:** 1 University Health Care Research Centre in Örebro County, Örebro, Sweden; 2 School of Health Sciences at Örebro University, Örebro, Sweden

**Keywords:** Distance, Interaction, Interpreting, Scoping review, Signed language, Telephone

## Abstract

**Background:**

Communication between people who are deaf and sign and people who use a spoken language is possible by means of an interpreter. Interpreting in real time can be performed at a distance, which differs from interpreting face-to-face. Due to COVID-19, interpretation at a distance has increased.

**Objective:**

The objective of this study was to map the existing literature to identify key characteristics by addressing the following question: What is known about interpreted mediated interactions between people using a signed respective spoken language across distances in real time?

**Design:**

Eight online databases, complemented by a search in one nonindexed journal of relevance to the review, were used to identify original studies published in 2010–2020, and 17 publications met the inclusion criteria. Charting of the data revealed insight from 17 original studies that were extracted, summarized, and reported.

**Results:**

Four key characteristics were identified: (1) advantages and challenges in remote interpreting; (2) the need for training in remote interpreting and video relay service (VRS); (3) regulations and organizational structures of VRS; and (4) the interpreter as an active party in VRS.

**Conclusion:**

Remote interpreting has several challenges but also advantages. Knowledge of these kinds of interactions is limited, and further research must be initiated and realized, not least due to technological developments and the increased number of interpreting events.

**Supplementary Information:**

The online version contains supplementary material available at 10.1186/s12913-022-07776-y.

## Background

Interpreting between spoken languages and signed languages at a distance has increased around the world due to restrictions resulting from COVID-19 [1]. Communication in real time between people who are deaf and signing and people who speak is made possible by an interpreter who signs for the deaf party what is said and says what the deaf party signs [2]. Interpreting between a spoken and a signed language may be performed simultaneously as the language modalities do not interfere, i.e., one modality is auditive while the other is visual [3].

For spoken languages, interacting in real time across distances via a telephone is not new as the telephone was patented in 1896 [4]. To communicate by telephone across spoken languages and physical distance is thus a matter of course for people who hear. For deaf people using signed languages, however, the situation has been different. Since signed languages rely on visual and gestural resources, interaction between two people using signed language is communicated through the video phone [5–7]. Video phone calls, i.e., telecommunication technologies between signers, became possible in the 1990s [8]. Currently, due to technical evolution, video calls are possible over the Internet and with different applications on smartphones (for example, Skype, Teams, Zoom, and Messenger).

Remotely interpreted interactions are found in various settings [9], such as in police hearings [10] and criminal proceedings [11, 12]. In these kinds of settings, communication between locations takes place via videoconference or some kind of video link-up system [9]. Remote interpreting is also used for conferences. In conferences interpreted remotely, there are two locations, and either the interpreter is in one of the two or all the parties are together in one location and the interpreter is in a separate, remote location [9].

Beyond different methods of remote interpreting, a service that performs interpreting between signed language and spoken languages at a distance in real time, *video relay service* (VRS), has become widespread [2, 13–16]. The parties in VRS are separated into three different locations, with the users of the service in two different locations and an interpreter in a call center. VRS is thus an interpreted phone call – a telecommunication technology - where the interpreter mediates between a party on a telephone and a party using a videophone or smartphone. VRS is organized and financed in various ways in the countries that provide this kind of service [17, 18].

Interpreter-mediated interactions in real time across distances differ from face-to-face interactions. In face-to-face situations, where all the people can see each other, they can interact using gazes, movements, and facial expressions that are not spoken or signed [19]. These kinds of resources cannot be used when people are physically apart from each other across distances. The COVID-19 pandemic has compelled people to engage in interactions across distances and remote interpreter-mediated interactions as well. As detailed in a research report by De Meulder, Pouliot, and Gebruers, K [1]., a total of 2634 sign language interpreters from 63 countries took part in a study of remote sign language interpreting during COVID-19. The study documented how the shift to remote interpreting has been experienced by and has impacted and prompted innovation in the sign language interpreting profession in ways that probably will endure beyond the pandemic. A majority of the respondents in one of the surveys reported that their remote workload was 0% in the last 6 months of 2019 (the beginning of the COVID-19 pandemic). A major shift in working practices was reported in April 2020: their jobs changed to 100% remote. Thus, a tremendous shift from face-to-face to remote interpreting occurred over only a couple of months. Despite the increasing numbers of interpreting at a distance, no reviews about this specific topic have been identified. However, there are reviews in related areas such as technical approaches to Chinese sign language processing [20], interpreting testing and assessment [21], terminology, taxonomy and key directions of distance interpreting according to spoken languages [22].

The current review was conducted to systematically map the research on interpreting at a distance as it differs from face-to-face interpreting and has increased. Remote interpreting is relatively new due to technical evolution. The following research question for this scoping review was formulated: What is known about interpreted mediated interactions between people using a signed respective spoken language across distances in real time? The objectives of the article were to map the existing literature to identify key characteristics of interpreter-mediated interactions in real time.

## Methods

The choice to conduct a scoping review for this study was based on the premise that a scoping review design is particularly suitable when the objective is to identify and systematically map the literature on a certain topic in a given research field [23]. Because no prior systematic mappings of interpreter-mediated interactions at distances have been identified, the scoping review methodology was deemed adequate to investigate this particular research area. Hence, in line with the methodology, the key characteristics of interpreter-mediated interactions in real time were explored.

The current study applies the original framework by Arksey and O’Malley [24], with further elaboration by Levac, Colquhoun, and O’Brien [25], Peters et al. [26], Peters et al. [27], and Peters et al. [28]. The framework contains five overarching steps: (1) *Identifying the research question:* Identifying, defining, and aligning the research question; (2) *Identifying relevant studies:* Developing and aligning inclusion and exclusion criteria; (3) *Study selection:* Selecting databases, search terms, and search strategies; (4) *Charting the data:* Deciding on the type of data to be extracted; and (5) *Collating, summarizing, and reporting the results*: Selecting, extracting, analyzing, and reporting the findings. In the current review, both of the authors were involved in every step during the entire process.

### Identifying the research question

Research on interpreting between signed and spoken languages in face-to-face interactions has increased [29]. With technical progress, interpreting between spoken and signed languages across distances has become a rapidly growing area of research [30]. Recently, the COVID-19 pandemic has required new ways of communicating and interpreting across distances. Real-time interpreting between a spoken and a signed language at a distance differs in several ways from interpreting in face-to-face situations. As a result, the rationales for this scoping review are: (1) Interpreting at a distance requires media (e.g., telephone, videophone, iPad) to make the interaction possible. Thus, the people involved must manage the technical components. (2) The used media affect the communication; i.e., cultural aspects influence the interaction. For example, signed conversation via videophone differs from signed or spoken conversations in face-to-face situations. In signed video conversations, people must adapt to the video cameras, which influence both articulation and articulation place [6]. Additionally, telephone conversations by hearing people differ from face-to-face interaction as such conversations rely exclusively on auditive resources as the interlocutors cannot see each other. (3) In interpreting at a distance, people who receive the interpretation cannot see each other and do not share physical space. Consequently, they must rely exclusively on the interpreter and cannot communicate with each other by nonverbal or gestural resources at all. The interpreter who is in contact with both of the users and the users of the interpretation must handle this matter.

To facilitate interpreter-mediated interactions at a distance, interpreters, users of the service, stakeholders, and designers (among others) need to have knowledge of how remote interactions are influenced in interpreter-mediated situations to enhance these interactions. To obtain this knowledge, the research question for the current review is: What is known about interpreted-mediated interactions between people using a signed respective spoken language across distance in real time? To answer this question, the existing literature was mapped and central characteristics of interpreted-mediated interactions between people using a signed respective spoken language across distance in real time were identified.

### Identifying relevant studies

By utilizing the population, concept, context (PCC) mnemonic, the following inclusion and exclusion criteria were set:

- *Population*: Interpreters who interpret between a spoken and a signed language and people who take part in the mediated interaction.
- *Concept:* To identify characteristics of research findings related to interpreter-mediated interactions across distances that involve interpreting between spoken and signed languages.
- *Context:* The interpreting setting should be related to simultaneous interpreting (i.e., where the signing person, the interpreter, and the speaking person are present but somehow physically separated).

Some other particulars were also set. The publication year for studies was set to between 2010 and 2020 based on the premise that the technology has developed rapidly and become more sophisticated during the last decade. Furthermore, few studies that fulfilled the inclusion criteria, published before 2010, were identified. Only original studies were accepted to capture only original scientific data, and the publication language had to be English because of the language skills of the authors of this study. Any kind of study design was accepted (i.e., both qualitative and quantitative).

#### Exclusion criteria

Studies that solely focused on technical aspects such as the design of technical equipment, non simultaneous interpreting (i.e., consecutive interpreting), and pre-recorded material (such as broadcasted interpreting) were excluded.

### Study selection

Based on the authors’ knowledge of the research area and to ensure a broad search and thereby identify relevant articles to include, eight databases were searched. These were CINAHL, Communication & Mass Media Complete, ERIC, Embase, Linguistics & Language Behavior Abstracts (LLBA), PubMed, Scopus, and Web of Science. Adequate search terms were identified by reading literature in the field and using the major headings connected to some of the databases (e.g., CINAHL headings). The search terms and search strategy varied among the databases due to differences in the database structures. All the searches were performed in cooperation with a scientific librarian. In addition, the *Journal of Interpretation* was searched manually as it was viewed as highly appropriate for the research question, albeit not indexed, in any of the above stated databases. In the Journal of Interpretation, the only search term used was “video” because the focus of that journal is signed language interpreting. The search was performed on two occasions: first in 2019 and then in January 2021. The search strategies for the databases are available in Additional file 1: Annex A. A complete list of search terms and database searches can be obtained from the first author.

### Charting the data

Prior to data extraction, a protocol for data charting was constructed and tested for feasibility. The protocol followed the PCC mnemonic to match the inclusion criteria. Information about the author/s, country, journal, scope (or objective/s), study design, and key characteristics were extracted and scheduled in an overview.

### Collating, summarizing, and reporting the results

In total, searches resulted in 3188 records from the databases and 42 from the *Journal of Interpretation*. After the duplicate check, 2502 records remained. By considering the inclusion and exclusion criteria, these records were screened at the title and abstract levels for inclusion by the two authors separately. When reading an article abstract, three judgments were possible: “yes,” “no,” or “maybe.” An abstract was deemed “maybe” if the information was unclear according to the inclusion criteria for the current study or if a record lacked an abstract. After the individual screening, the two authors’ decisions were compared and any disagreements resolved through discussion. Abstracts deemed “yes” or “maybe” by the two authors in consensus were retrieved in full text and assessed for eligibility (*n* = 72).

**Fig. 1:**
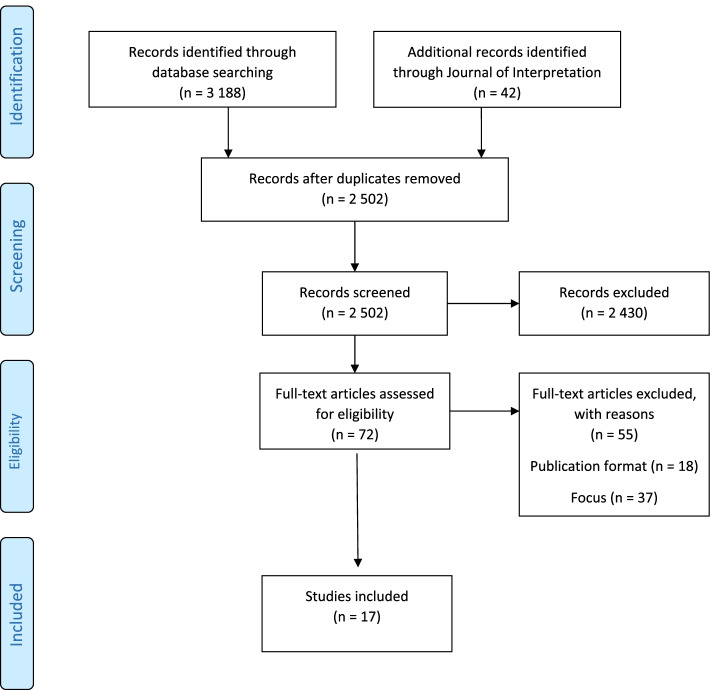
The inclusion process based on PRISMA flow chart (Moher et al. [31])

After the full text articles were read, 55 were excluded. Articles were excluded either because the studies had the wrong focus (e.g., broadcasted remote interpreting on television) (*n* = 37) or because the publication format did not match the inclusion criteria for type of publication (e.g., theses/dissertations, book chapters, conference abstracts) (*n* = 18). As in the previous step, the full text papers were read and judged by the two authors separately, and individual judgments were compared and discussed. Ultimately, 17 papers were deemed adequate for this scoping review. A flow chart of the inclusion process is presented in Fig. 1.

The two authors retrieved the information in the included articles separately. Following the previous steps, the information was compared and discussed to ensure reliability in the data charting process. The key characteristics were analyzed narratively in a summarized form by using headings and subheadings, which were constructed based on the content of the main findings.

## Results

In total, 17 studies fulfilled the inclusion criteria and were included in this scoping review (Table 1).

**Table 1:** Overview of included studies

Author(s)	Year	Scope	Study design
Alley, E [32].	2014	To investigate the origins of VRS guidelines by reviewing public documents from the Federal Communications Commission and the Americans with Disabilities Act and interpreters’ perceptions of constraints and influences on VRS work.	Interviews with interpreters (n = 4); purposeful sampling; open and closed coding process
Bower, C [33].	2015	To illuminate interpreters’ experiences with stress and burnout in VRS interpreting and to provide ideas to solve this problem.	Survey (*n* = 395 full responses) snowball sampling
Ehrlich, S. & Vance, K [34].	2015	To explore the concept of direct versus indirect on-demand interpreting, with the goal of developing a framework of considerations when offering on-demand interpreting services.	Case study, pre- and post-survey with the student and the supervisor; convenience sampling
Haualand, H [35].	2014	To discuss how the multiple definitions and ways of organizing videophones within three sociotechnical systems (Norway, Sweden, and the USA) mediate agency and the resulting implications for inclusion and accessibility.	Field study: observations and interviews (formal and informal; *n* = not defined)
Kushalnagar, P., Paludneviciene, R., & Kushalnagar, R [36].	2019	To investigate the national trends in deaf patients’ satisfaction with the quality of video remote interpreting in healthcare settings and recommend actions to improve video remote interpreting quality and deaf patients’ satisfaction with it in healthcare settings.	Survey, initial survey (*n* = 968), final user sample (*n* = 555)
Marks, A [37].	2018	To describe and analyze features of turn management in American Sign Language/ English video relay interpreted calls.	Simulated VRS calls (*n* = 2), sociolinguistic analysis
Napier, J., & Leneham, M [38].	2011	To investigate whether the use of current technology within the New South Wales (Australia) Department of Justice is appropriate for providing video remote sign language interpreting.	Quasi-experimental design: observation and follow-up interviews; testing five scenarios using scripts
Napier, J., Skinner, R., & Turner, G [39].	2017	To identify common issues facing interpreters when working in remote environments and to ascertain what aspects of interpreting remotely via a video link are working successfully.	Survey of interpreters from 16 different countries (*n* = 58)
Palmer, J.L., Reynolds, W., & Minor, R [40].	2012	To examine how the use of videophones in the USA affects American Sign Language and what role VRS interpreters play in potential language standardization.	Triangulation: Qualitative; 2 focus groups with 4 participants in each group (one group with deaf VRS users and the second with VRS interpreters) Quantitative; one survey for deaf consumers (*n* = 81) and one survey for interpreters (*n* = 131)
Roman, G.A., & Samar, V [41].	2015	To identify the parts of the body where video interpreters experience the most musculoskeletal pain and to determine whether ergonomic knowledge is associated with improvements in posture and decreases in complaints of musculoskeletal pain.	Mixed method; pre-treatment survey for interpreters (*n* = 101), pre-and post-investigation of ergonomic knowledge, posture quality and self-reported pain after a workshop; intervention group (*n* = 78), control group (*n* = 23)
Treviño, R., & Quinto-Pozos, D [42].	2018	To investigate how trilingual (American Sign Language, English, and Spanish) interpreters pronounce names that commonly appear in either English or Spanish phonology.	Experimental; demographic questionnaire; two mock videophone calls; semi-structured interviews (in total *n* = 20)
Turner, G.H., Napier, J., Skinner, R., & Wheatley, M [43].	2017	To investigate how differences in telecommunication relay in Europe give rise to particular issues affecting how and when deaf people can access and use telecommunication relay and then to identify the perceived advantages and disadvantages of telecommunication relay.	Mixed method; online survey (*n* = 84), follow-up interviews (*n* = 7)
Warnicke, C., & Plejert, C [44].	2012	To describe, analyze, and discuss how patterns of turn-taking are administered in Swedish VRS, with a particular focus on techniques and strategies used by the interpreter to manage interactions.	Conversation analysis (CA) of authentic recordings (*n* = 13)
Warnicke, C., & Plejert, C [45].	2016	To explore the interpreter’s positioning (i.e., how interpreters orient themselves toward interactions) in Swedish VRS on a moment-to-moment basis.	CA of authentic recordings (*n* = 13)
Warnicke, C., & Plejert, C [15].	2018	To explore how the interpreter is oriented toward the headset, turning it into an interactional resource.	CA of authentic recordings (*n* = 13)
Wessling, D.M., & Shaw, S [46].	2014	To understand how VRS interpreters perceive their ability to cope with extremely traumatic call content.	Online survey (*n* = 889) closed and open question for interpreters
Yabe, M. [47]	2020	To identify healthcare providers and deaf/hard of hearing patients’ preferences for remote respective in-person interpreting during critical care and noncritical care.	Online survey: healthcare providers who primarily worked with deaf/hard of hearing patients (*n* = 26), and deaf/hard of hearing patients (*n* = 41) Interviews: healthcare providers (*n* = 8), deaf/hard of hearing patients (*n* = 8)

The included studies were retrieved from eleven different scientific journals, with most studies identified in the *Journal of Interpretation* (*n* = 4), *Translation and Interpreting* (*n* = 2) and *Translation and Interpreting Studies* (*n* = 2). Almost half of the studies (*n* = 8/9) were carried out in the USA (Table 2).

**Table 2:** Study characteristics

Study characteristics	***n*** = 17
***Journal***	
Disability and Health Journal	1
Ethnos	1
Information, Communication & Society	1
Interpreting	2
Interpreters Newsletter	1
Journal of Interpretation	4
Journal of Medical Internet Research (JMIR)- Rehabilitation Assistive Technologies	1
Journal of Pragmatics	1
Sign Language Studies	1
Translation and Interpreting	2
Translation and Interpreting Studies	2
***Country/region***	
Australia	1
Canada	1
Europe	1
Norway	1
Sweden	3
UK	1
USA	8
USA & Canada	1

In the current review, four key characteristics were identified and reported related to the research question of what is known about interpreter-mediated interactions between people using a signed respective spoken language across distances in real time. The key characteristics were: (1) *the advantages and challenges in remote interpreting;* (2) *the need for training in both remote interpreting and VRS;* (3) *regulations and organizational structures of VRS*; (4) *the interpreter as an active party in VRS.*

### Advantages and challenges in remote interpreting

Remote interpreting—when some of the parties at an interpreted event are located remotely—gives people immediate access to interpreting, which is beneficial for the deaf community as it can be difficult to obtain an interpreter at all [39]. Thus, remote interpreting offers an alternative to receive interpreting. Promptness is one positive aspect that was stressed by providers in acute situations in health care settings in a study by [47]. The patients in that study did not all express the same amount of satisfaction with remote interpreting, however, although they reported that they could accept remote interpreting in health care situations if there were specific reasons for it. The patients pointed out that remote interpreting might be suitable for follow-up situations—especially for noncritical appointments [47].

Remote interpreting has been observed and tested in several settings, such as school [34] and courtrooms [38]. It has been evaluated in healthcare settings [36, 47] and investigated from an interpreter’s perspective [39]. One positive aspect from interpreters’ standpoint is saved travel time and the possibility of working from home [39]. Regarding the use of educational interpreting via the iPad, a positive impact has been reported on individual performance in the workplace [34].

Although there are some advantages, studies involving observed remote interactions have reported several challenges in interpreting settings concerning technological, linguistic, environmental, and logistical issues. From a technological perspective, access to consistent and stable Wi-Fi is a challenge in numerous settings [34, 38]. In remote settings, the screen can be a limitation in several aspects. Because signed languages are three-dimensional languages, based on visual and gestural resources and rendered in a two-dimensional form in remote settings, the screen can make it difficult to decode what is signed [38]. Additionally, the background at the interlocutor’s location may be distracting for both the signing party and the interpreter as they attempt to see what is signed [38]. Varying sizes of television screens—especially when a screen is divided into smaller sections to display different images—make it even more difficult as it can be difficult to obtain a clear idea of who is speaking and to decode what is fingerspelled as production/readback must be adjusted to enhance visibility and clarity [38]. An interactive challenge is the lack of patient-provider relationships [47]. Attracting other people’s attention when needed in an interpreted event performed remotely is another relative challenge as there are limited opportunities to establish cues to gain attention prior to the event, such as in (mock) trials [38].

When comparing face-to-face with remote interpreting, both (hearing) professionals and deaf patients prefer interpreters in person to facilitate effective communication and translation accuracy and thereby enable better treatment in healthcare settings [47]. Evaluations of remote interpreting among deaf adults who received interpreting services in a healthcare setting showed that those who had a healthcare provider whom they visited regularly were significantly more dissatisfied with the remote interpreting services compared to respondents who did not have a regular provider [36]. In sum, according to the studies on remote interpreting included in the current review, there seem to be few advantages but several challenges with remote interpreting.

### Need for training on both remote interpreting and VRS

Because remote interactions differ from face-to-face interpreting, formal training has been suggested as a way to improve interactions [47]. Interpreters note that education programs have a responsibility to prepare students for remote interpreting in VRS [32] and that VRS and remote interpreters need training appropriate to the actual setting [39]. However, what special kind of training has not been specified.

Ergonomic training among video interpreters is one aspect that has been researched. The results of ergonomic workshops for video interpreters have significantly enhanced ergonomic knowledge: in one study, those with little ergonomic knowledge prior to a workshop improved their knowledge the most, and the interpreters who took part reported feeling less pain after the workshop [41]. Moreover, it is stated that customers who use a given service need training [47]. Training on customer service skills is needed to improve interpreting services to make users aware of how to best use VRS and remote interpreting [39]. Although the question of training and education is addressed, only one study [41] reports on how training or education has been provided or organized.

### Regulations and organizational structures of VRS

VRS is a service that provides interpreted calls from a call center. Studies on VRS show that regulations and organization differ around the world, although they may appear the same on the surface [35, 43]: The service is recognized as a call for functional equivalence in the USA, a telecommunication service provided by interpreters in Sweden, and an exclusive network extending the Sign Language-Interpreting service in Norway [35]. Country-specific VRS has dissimilar origins. It also has dissimilar goals, as it is related to politics and financial conditions [32, 35, 43]. Investigations of the systems in the USA, Sweden, and Norway reveal the influence of organization and regulations: The service is recognized as a call for functional equivalence in the USA, a telecommunication service provided by interpreters in Sweden. In contrast, the Norwegian service is organized as an external service and is not a part of the national telecom service [35].

In the US, interpreters must abide by both government regulations and the economics of the companies involved [32]. Given the need to keep both these regulations and economic aspects of the providing companies in mind, problems with VRS provider policies have been reported [33]. Interpreters have reported feeling uncertain about federal regulations and company rules and that they have become “non-people,” without the ability to control and manage their work [32].

However, those who use VRS service would welcome a pan-European, multilingual telecommunication relay service, especially to remove barriers to contacting European institutions [43]. There is thus some desire for an organized (broader) service from the users’ perspective. However, different countries have their own goals and regulations, which in some cases are vague for interpreters.

### Interpreter as an active part in VRS

The identified studies mostly focus on interpreters, their performance, and their working conditions. Of the studies included in the current review, eleven concern VRS. It is apparent that interpreters have a special status in interactions. In the sections below, three categories related to interpreters as active parties in these settings are presented: (1) *role and responsibility*; (2) *language use*; and (3) and *health and working conditions*.

#### Role and responsibility

Interpreters have a special status and are central to VRS as they may be the only interpreters who are directly linked to the users of VRS. This implies that the interpreter is obligated to interpret and control the situation to facilitate common ground among all the parties [45]. Interpreters’ several tasks in VRS align with their responsibility to provide information about the service so that the interlocutors understand the permission requirements of the setting and the interactions of the call [45]. The interpreter decides what is and is not communicated [44]. Given the interpreter’s strong influence on a call, it is challenging when the interpreter does not obtain information or cannot prepare before the interpreting event [32, 37, 38, 44, 45, 48]. A lack of preparation is problematic and becomes relevant in relation to the interpreting as the changes between different contexts is faced in every single call [43]. In a moment-to-moment basis, lack of preparation becomes challenging as the technology makes limitations to decode, for example, digits and numbers [35, 43]. Preparation does not generate revenue for service providers (in the USA), which puts pressure on interpreters [32]. Interpreters’ role and responsibility are affected as interpreters must adapt to the setting [45] and at the same time take (different) regulations into account when working in VRS [32].

#### Language use

In VRS, the caller does not know which interpreter will answer the call, and the interpreter answering the call does not know who is calling beforehand. This uncertainty can affect both which signs to use and how to pronounce things. Interpreters have reported that it can be difficult to acknowledge deaf people’s regional variations [40] and that it can be challenging to pronounce names in trilingual settings [42]. In VRS, the caller and the called party are dependent on the interpreter, and interpreters may use code blending (i.e., partly speaking and signing at the same time to enhance understanding of what is occurring for both parties) [45]. Turn organization — which interpreters must handle in VRS — is difficult as none of the users of the service can see or hear each other [37, 44]. The interpreter may use strategies such as anticipating upcoming utterances, synchronizing utterances, expanding renditions, and reducing renditions to manage turn organization [44]. These strategies have been identified in interpreted interactions during regular calls in Sweden [44]; similar strategies have been found in simulated VRS calls in the USA [37]. There are also identified techniques that are not part of renditions. These techniques seem similar in both Sweden [44] and the USA [37] and comprise hand and body movements, gazes and audible signals, and definitions of the situation [44].

In the VRS setting, the interpreter wears a headset to listen to the speaking party. The headset is used as an interactional resource and facilitates interactions among all the parties on the VRS call in contact with the signing party [15, 37]. The interpreter may point to the headset, hold the headset, or navigate toward the headset to organize the interactions in the visual arena [15]. Thus, the setting affects how language is used in several ways in VRS.

#### Health and working conditions

Remote interpreting affects interpreters’ health and working conditions. In VRS, one difference from working in a face-to-face setting is that assignments turn around at a fast pace [32, 46]. Interpreters say they must cope with users’ emotional extremes [46]. The reported emotions are mostly negative, such as anger, sadness, and frustration, although positive emotions, such as happiness, have been reported [46]. Interpreters’ opportunity to report coping responses occurs after the assignment is complete [46]. This implies that due to a lack of breaks and recovery time, as in VRS, coping responses and control are rarely possible. Other documented stress factors in VRS include (among others) managing calls in which the caller is angry with the interpreter, concern about the length of time between calls, receiving a 911 call, concern about physical strain, and interpreting calls with limited contextual information [33]. These stress factors are related to actual calls and users of the service and may be difficult to avoid or to handle. To reduce stress, interpreters suggest that overall efforts be made at the organizational level such as reduced call volume, increased break time, greater flexibility with policies regarding statistical requirements, more opportunities for teams and debriefings (support), and improvements to management [33]. Additionally, technical improvements, such as better audio/video quality and better network reliability, would help [39]. Thus, interpreters recommend a clearer organizational structure that could improve their working conditions in VRS settings. Uncertainty is a recurring theme related to interpreters’ working conditions in remote settings. In VRS, there is uncertainty about what type of call is coming next, the context of the call, and the users’ signing style [33, 39]. In both VRS and courtroom settings, a lack of time for preparation and difficulties with technology complicate the situation for interpreters [33, 39]. Pain [41], stress, and burnout among interpreters are recurrent effects reported in VRS [33].

## Discussion

The current study centers on interpreter-mediated interactions between people using a signed respective spoken language across distances in real time. All the included studies were explorative by nature. None of the identified studies applied an accumulating or follow-up design. This is not surprising since the research area is quite new. However, there is obvious a need for scientific studies of interpreting at a distance. Practice needs to be explored from different aspects of interpreting, interaction and performance to broadening the landscape of interpreting between signed and spoken language at a distance. The key findings revealed many facets that could be highlighted in practice and in future research. For instance, interpreting across distances creates a different kind of interaction from interpreting in a face-to-face setting. The physical separation and the media place new demands on the situation and on the interlocutors. One might assume that adding a certain technique to interpreting at a distance could resolve issues of accessibility. According to the studies examined in the current review, new challenges are emerging in interpreting across distances. Nonetheless, improved technical developments have probably improved the conditions for remote interpreting, since some challenges are linked to video equipment. Concerning the COVID-19 pandemic, interpreting across distances could be a solution, although research on interpreting in the context of this pandemic is limited. The pandemic has driven up the number of interpreter-mediated events at a distance but also those of other kinds of remote interactions. This may lead to improved conditions, such as better technology and easier handling of the technology. The studies in this scoping review are from 2010 onward, and both technology and its use have probably developed over time. Some challenges with the technology may have already decreased and been resolved.

Most studies have been carried out in the USA. This is not surprising given that the USA is a large Western country with considerable technological development. As in many other Western nations, deaf persons in the USA are, based on the Americans with Disabilities Act (ADA), entitled to the same rights as any other civilian, including the right to adequate communication in society and during interactions with authorities and civic services. Consequently, VRS interpreting is a developed service in the USA; unsurprisingly, research has appeared on this topic. In some countries with a limited number of interpreters, however, it can be dangerous, difficult, expensive, or far for interpreters to travel to an assignment. In these countries, remote interpreting may save time, costs, and even lives. For sufficient conditions for remote interpreting, technical equipment is needed, such as stable internet and computers for both interpreters and signing users. These aspects can be challenging in less developed nations.

From the findings, it is also clear that remote interpreting may require a specific focus in interpreter education. Aspects such as ergonomics and other physical health issues may be overlooked in traditional training because they are viewed as non-issues in face-to-face interpreting. Furthermore, uncertainty over the impossibility of preparing for incoming calls in VRS is highlighted as a stressor for interpreters resulting in little control of their work situation. In the general literature on working-life, little control over one’s work is considered a major risk of ill health and can subsequently result in sick leave [49]. It may therefore be necessary to ask whether VRS interpreters are at greater risk than other interpreters in terms of ill health and, in the long run, sick leave.

In the current review, the consumer view on interpreting services at a distance is missing. The current trend, to redirect interpreting from face-to-face interaction to mediation at a distance may be challenging for the persons who utilize the service. One important issue that raises relates to financial aspects: will economic benefits for the companies limit deaf (and hearing) people’s possibility to choose between interpreting face-to-face versus at a distance? Will stakeholders prioritise interpreting at a distance without consider consequences for interpreter’s and consumers of interpreting services? What forces will prevail over forthcoming organization of interpreting services?

### Limitations of the scoping review methodology

In the current review, no quality assessments were made of the included studies. This might be viewed as a limitation. However, when investigating an area where no previous reviews have been undertaken, the first step must be to simply map the literature to identify “what has been done”. There is limited research in this area, and it was considered important to report the findings from all studies. Although no quality assessments were performed, four key characteristics emerged based on the included studies. Hopefully, these characteristics can guide researchers conducting future research in this area and guide educators in addressing remote interpreting and VRS interpreting in education for interpreters, users and stakeholders.

## Supplementary Information

**Additional file 1: Appendix A.** Search strategies in the databases.

## Data Availability

See Additional file 1: Annex A.
