# Supplementary material for: Interpreter-mediated interactions between people using a signed respective spoken language across distances in real time: a scoping review
Source: BMC Health Serv Res. 2022 Mar 24;22:387. doi: 10.1186/s12913-022-07776-y (PMC8943107; doi:10.1186/s12913-022-07776-y)
Supplement: Supplementary file 1 — Additional file 1: Appendix A. Search strategies in the databases. [file 12913_2022_7776_MOESM1_ESM.docx]

Appendix A. Search strategies in the databases

PubMed

| Search terms | | | Identified records |
| --- | --- | --- | --- |
|  | | | |
|  | 1 | "Translating"[Mesh] |  |
|  | 2 | (translat*[Title/Abstract] OR interpret*[Title/Abstract]) |  |
|  | 3 | 1 OR 2 |  |
|  | 4 | ("Computers"[Mesh] OR "Telecommunications"[Mesh]) |  |
|  | 5 | (telephone*[Title/Abstract] OR phone*[Title/Abstract] OR videophone*[Title/Abstract] OR smartphone*[Title/Abstract] OR mobile*[Title/Abstract] OR video*[Title/Abstract]) |  |
|  | 6 | 4 OR 5 |  |
|  | 7 | ("Sign Language"[Mesh] OR "Hearing Disorders"[Mesh] OR "Deaf-Blind Disorders"[Mesh] OR "Persons With Hearing Impairments"[Mesh]) |  |
|  | 8 | ("sign language"[Title/Abstract] OR "signed language"[Title/Abstract] OR "hearing disorder"[Title/Abstract] OR "hearing disorders"[Title/Abstract] OR deaf*[Title/Abstract] OR "hearing impairment"[Title/Abstract] OR "hearing impairments"[Title/Abstract] OR "auditory impairment"[Title/Abstract] OR "auditory impairments"[Title/Abstract] OR "hard of hearing"[Title/Abstract] OR "cued speech"[Title/Abstract]) |  |
|  | 9 | 7 OR 8 |  |
|  | 10 | 3 AND 6 AND 9 | 184 |

ERIC (Ebsco)

| Search terms | | | Identified records |
| --- | --- | --- | --- |
|  | | | |
| 1 |  | DE "Sign Language" OR DE "American Sign Language" OR DE "Cued Speech" OR DE "Hearing Impairments" OR DE "Deafness" OR DE "Partial Hearing" OR DE "Deaf Blind" |  |
| 2 |  | "sign language" OR "signed language" OR "hearing disorder*" OR deaf* OR "hearing impairment*" OR "auditory impairment*" OR "hard of hearing" OR "cued speech" |  |
| 3 |  | S1 OR S2 |  |
| 4 |  | DE "Deaf Interpreting" |  |
| 5 |  | \| interpret* OR translat* \|  \| \| --- \| --- \| |  |
| 6 |  | \| S4 OR S5 \|  \| \| --- \| --- \| |  |
| 7 |  | DE "Telecommunications" OR DE "Computer Mediated Communication" OR DE "Radio" OR DE "Teleconferencing" OR DE "Television" OR DE "Teleconferencing" OR DE "Videoconferencing" OR DE "Computer Mediated Communication" OR DE "Electronic Mail" OR DE "Electronic Publishing" OR DE "Social Media" OR DE "Computers" OR DE "Laptop Computers" OR DE "Handheld Devices" OR DE "Computer Use" OR DE "Social Media" OR DE "Virtual Classrooms" OR DE "Virtual Universities" OR DE "Web 2.0 Technologies" |  |
| 8 |  | video* OR telephone* OR phone* OR videophone* OR smartphone* OR mobile* |  |
| 9 |  | \| S7 OR S8 \|  \| \| --- \| --- \| |  |
| 10 |  | S3 AND S6 AND S9 | 231 |

CINAHL Plus with Full Text

| Search terms | | | Identified records |
| --- | --- | --- | --- |
|  | | | |
| 1 |  | (MH "Sign Language") OR (MH "Hearing Disorders+") |  |
| 2 |  | "sign language" OR "signed language" OR "hearing disorder*" OR deaf* OR "hearing impairment*" OR "auditory impairment*" OR "hard of hearing" OR "cued speech" |  |
| 3 |  | S1 OR S2 |  |
| 4 |  | (MH "Interpreter Services") |  |
| 5 |  | interpret* OR translat* |  |
| 6 |  | S4 OR S5 |  |
| 7 |  | (MH "Telecommunications+") OR (MH "Audiovisuals+") OR (MH "Videorecording+") OR (MH "Computers and Computerization+") |  |
| 8 |  | video* OR telephone* OR phone* OR videophone* OR smartphone* OR mobile* |  |
| 9 |  | S7 OR S8 |  |
| 10 |  | S3 AND S6 AND S9 | 346 |

Communication and Mass Media Complete (Ebsco)

| Search terms | | | Identified records |
| --- | --- | --- | --- |
|  | | | |
| 1 |  | ((DE "TELECOMMUNICATION" OR DE "ART & telecommunication" OR DE "ARTIFICIAL satellites in telecommunication" OR DE "BANDLIMITED communication" OR DE "BANDWIDTHS" OR DE "BROADCASTING industry" OR DE "CABLE telecommunication services" OR DE "CHAOTIC communication" OR DE "COMMUNICATION infrastructure" OR DE "COMMUNICATION revolution" OR DE "COMPUTER networks" OR DE "CONVERGENCE (Telecommunication)" OR DE "CROSSTALK" OR DE "DIGITAL communications" OR DE "IMAGE transmission" OR DE "INDOOR communications (Telecommunication)" OR DE "INTERACTIVE voice response (Telecommunication)" OR DE "INTERSTELLAR communication" OR DE "MACHINE-to-machine communications" OR DE "MEDIA spillover" OR DE "MILITARY telecommunication" OR DE "MOLECULAR communication (Telecommunication)" OR DE "MULTICHANNEL communication" OR DE "MULTIPLEXING" OR DE "NETWORK performance" OR DE "OPTICAL communications" OR DE "PHOTOTELEGRAPHY" OR DE "PODCASTING" OR DE "RADIO (Medium)" OR DE "RADIO telemetry" OR DE "REMOTE broadcasting" OR DE "RURAL telecommunication" OR DE "SATELLITE radio services" OR DE "SEMICONDUCTOR lasers in telecommunication" OR DE "SHARED tenant services (Telecommunication)" OR DE "SIGNAL theory" OR DE "SPEECH processing systems" OR DE "SPEECH scramblers" OR DE "SPREAD spectrum communications" OR DE "TELECOMMUNICATION -- Social aspects" OR DE "TELECOMMUNICATION channels" OR DE "TELECOMMUNICATION in business" OR DE "TELECOMMUNICATION in education" OR DE "TELECOMMUNICATION links" OR DE "TELECOMMUNICATION systems" OR DE "TELECOMMUNICATIONS services" OR DE "TELECONFERENCING" OR DE "TELEGRAPH & telegraphy" OR DE "TELEMATICS" OR DE "TELEPHONE call processing" OR DE "TELEPHONES" OR DE "TELEPORTS (Telecommunication)" OR DE "TELEVISION" OR DE "TRANSPONDERS" OR DE "UNDERGROUND communication (Telecommunication)" OR DE "VIDEO on demand" OR DE "VIRTUAL communications" OR DE "VIRTUAL communities" OR DE "VIRTUAL networks" OR DE "WEBCASTING" OR DE "WIRELESS telecommunication services industry" OR DE "WIRELESS telegraph") OR (DE "TELEPHONES" OR DE "CABLE telephone services" OR DE "CALLER ID telephone service" OR DE "CELL phones" OR DE "CORDLESS telephones" OR DE "CROSSTALK" OR DE "PUBLIC telephones" OR DE "RADIOTELEPHONE" OR DE "TELEPHONE & youth" OR DE "TELEPHONE call processing" OR DE "TELEPHONE calls" OR DE "TELEPHONE numbers" OR DE "VIDEO telephones")) OR (DE "COMPUTER conferencing" OR DE "INTERNET videoconferencing" OR DE "WEBINARS") |  |
| 2 |  | video* OR telephone* OR phone* OR videophone* OR smartphone* OR mobile* |  |
| 3 |  | 1 OR 2 |  |
| 4 |  | ((DE "TRANSLATING & interpreting" OR DE "FRENCH language -- Translating" OR DE "GERMAN language -- Translating" OR DE "HEBREW language -- Translating" OR DE "ITALIAN language -- Translating" OR DE "JAPANESE language -- Translating" OR DE "LATIN language -- Translating" OR DE "OLD English language -- Translating" OR DE "OPERA translating" OR DE "PUBLIC service interpreting" OR DE "SELF-translation" OR DE "SPANISH language -- Translating" OR DE "TRANSLATING of Greek language") OR (DE "INTERPRETERS for the deaf")) |  |
| 5 |  | interpret* OR translat* |  |
| 6 |  | 4 OR 5 |  |
| 7 |  | (((DE "SIGN language" OR DE "AMERICAN Sign Language" OR DE "BABY signing (Sign language)" OR DE "BRITISH Sign Language" OR DE "CZECH Sign Language" OR DE "ESTONIAN Sign Language" OR DE "FRENCH Belgian Sign Language" OR DE "GREEK Sign Language" OR DE "HUMOR in sign language" OR DE "KOREAN Sign Language" OR DE "MAURITIAN Sign Language" OR DE "PAKISTAN Sign Language") OR (DE "MEANS of communication for deaf people" OR DE "CUED speech" OR DE "DEAF -- Speech" OR DE "FINGER spelling" OR DE "LIPREADING")) OR (DE "HEARING impaired" OR DE "DEAF" OR DE "HEARING impaired children")) OR (DE "HEARING disorders" OR DE "AUDITORY neuropathy" OR DE "AUDITORY processing disorder" OR DE "DEAFNESS" OR DE "HYPERACUSIS" OR DE "PENDRED syndrome" OR DE "TINNITUS" OR DE "TELECOMMUNICATIONS devices for the deaf") |  |
| 8 |  | "sign language" OR "signed language" OR "hearing disorder*" OR deaf* OR "hearing impairment" OR "auditory impairment" OR "hard of hearing" OR "cued speech" |  |
| 9 |  | 7 OR 8 |  |
| 10 |  | 3 AND 6 AND 9 | 394 |

Embase.com

| Search terms | | | Identified records |
| --- | --- | --- | --- |
|  | | | |
| 1 |  | 'sign language'/exp OR 'hearing disorder'/exp OR 'hearing impaired person'/exp |  |
| 2 |  | 'sign language':ti,ab,kw OR 'signed language':ti,ab,kw OR 'hearing disorder*':ti,ab,kw OR deaf*:ti,ab,kw OR 'hearing impairment*':ti,ab,kw OR 'auditory impairment*':ti,ab,kw OR 'hard of hearing':ti,ab,kw OR 'cued speech':ti,ab,kw |  |
| 3 |  | #1 OR #2 |  |
| 4 |  | 'interpreter service'/exp |  |
| 5 |  | interpret*:ti,ab,kw OR translat*:ti,ab,kw |  |
| 6 |  | #4 OR #5 |  |
| 7 |  | 'telecommunication'/exp OR 'telephone'/exp OR 'social media'/exp OR 'mobile phone'/exp OR 'videoconferencing'/exp OR 'wireless communication'/exp OR 'computer'/exp OR 'computer interface'/exp OR 'mobile application'/exp OR 'web browser'/exp OR 'videorecording'/exp OR 'webcast'/exp |  |
| 8 |  | video*:ti,ab,kw OR telephone*:ti,ab,kw OR phone*:ti,ab,kw OR videophone*:ti,ab,kw OR smartphone*:ti,ab,kw OR mobile*:ti,ab,kw |  |
| 9 |  | #7 OR #8 |  |
| 10 |  | #3 AND #6 AND #9 | 290 |

Web of Science (Indexes=SCI-EXPANDED, SSCI, A&HCI, CPCI-S, CPCI-SSH, ESCI)

| Search terms | | | Identified records |
| --- | --- | --- | --- |
| 1 |  | TS=("sign language" OR "signed language" OR "hearing disorder*" OR deaf* OR "hearing impairment*" OR "auditory impairment*" OR "hard of hearing" OR "cued speech") |  |
| 2 |  | TS=(interpret* OR translat*) |  |
| 3 |  | TS=(video* OR telephone* OR phone* OR videophone* OR smartphone* OR mobile*) |  |
| 4 |  | #3 AND #2 AND #1 | 501 |

Scopus

| Search terms | | | Identified records |
| --- | --- | --- | --- |
|  | | | |
| 1 |  | TITLE-ABS-KEY ( ( "sign language" OR "signed language" OR "hearing disorder*" OR deaf* OR "hearing impairment*" OR "auditory impairment*" OR "hard of hearing" OR "cued speech" ) ) |  |
| 2 |  | TITLE-ABS-KEY ( ( interpret* OR translat* ) ) |  |
| 3 |  | TITLE-ABS-KEY ( ( video* OR telephone* OR phone* OR videophone* OR smartphone* OR mobile* ) ) |  |
| 4 |  | #1 AND #2 AND #3 | 927 |

Linguistics and Language Behavior Abstracts (LLBA)

| Search terms | | | Identified records |
| --- | --- | --- | --- |
|  | | | |
| 1 |  | MAINSUBJECT.EXPLODE("Sign Language") OR MAINSUBJECT.EXPLODE("Cued Speech") OR MAINSUBJECT.EXPLODE("Hearing Disorders") |  |
| 2 |  | noft("sign language" OR "signed language" OR "hearing disorder*" OR deaf* OR "hearing impairment*" OR "auditory impairment*" OR "hard of hearing" OR "cued speech") |  |
| 3 |  | S1 OR S2 |  |
| 4 |  | MAINSUBJECT.EXPLODE("Translation") |  |
| 5 |  | noft(interpret* OR translat*) |  |
| 6 |  | S4 OR S5 |  |
| 7 |  | MAINSUBJECT.EXPLODE("Telephone") OR (MAINSUBJECT.EXPLODE("Interactive Video") OR MAINSUBJECT.EXPLODE("Videoconferencing")) OR MAINSUBJECT.EXPLODE("Computer Mediated Communication") OR (MAINSUBJECT("Computer Applications") OR MAINSUBJECT("Computer Assisted Instruction")) |  |
| 8 |  | noft(video* OR telephone* OR phone* OR videophone* OR smartphone* OR mobile*) |  |
| 9 |  | S7 OR S8 |  |
| 10 |  | S3 AND S6 AND S9 | 315 |
